# Plant Growth Promotion Potential Is Equally Represented in Diverse Grapevine Root-Associated Bacterial Communities from Different Biopedoclimatic Environments

**DOI:** 10.1155/2013/491091

**Published:** 2013-06-26

**Authors:** Ramona Marasco, Eleonora Rolli, Marco Fusi, Ameur Cherif, Ayman Abou-Hadid, Usama El-Bahairy, Sara Borin, Claudia Sorlini, Daniele Daffonchio

**Affiliations:** ^1^Department of Food, Environment, and Nutritional Sciences, University of Milan, Via Celoria 2, 20133 Milan, Italy; ^2^Laboratory of Microorganisms and Active Biomolecules, University of Tunis El Manar, Campus Universitaire, Rommana 1068, Tunis BP 94, Tunisia and Laboratory BVBGR, ISBST, University of Manouba, La Manouba 2010, Tunisia; ^3^Department of Horticulture, Faculty of Agriculture, Ain Shams University, Shubra Elkheima, Cairo, Egypt

## Abstract

Plant-associated bacteria provide important services to host plants. Environmental factors such as cultivar type and pedoclimatic conditions contribute to shape their diversity. However, whether these environmental factors may influence the plant growth promoting (PGP) potential of the root-associated bacteria is not widely understood. To address this issue, the diversity and PGP potential of the bacterial assemblage associated with the grapevine root system of different cultivars in three Mediterranean environments along a macrotransect identifying an aridity gradient were assessed by culture-dependent and independent approaches. According to 16S rRNA gene PCR-DGGE, the structure of endosphere and rhizosphere bacterial communities was highly diverse (*P* = 0.03) and was associated with a cultivar/latitudinal/climatic effect. Despite being diverse, the bacterial communities associated with Egyptian grapevines shared a higher similarity with the Tunisian grapevines than those cultivated in North Italy. A similar distribution, according to the cultivar/latitude/aridity gradients, was observed for the cultivable bacteria. Many isolates (23%) presented *in vitro* multiple stress resistance capabilities and PGP activities, the most frequent being auxin synthesis (82%), insoluble phosphate solubilisation (61%), and ammonia production (70%). The comparable numbers and types of potential PGP traits among the three different environmental settings indicate a strong functional homeostasis of beneficial bacteria associated with grape root.

## 1. Introduction

Grapevine is among the most ancient crops grown in the Mediterranean basin. Historically, grape and derived products held an important economic role in the area, and this importance persists to the present day. Vineyards are extremely sensitive to phytopathogen attacks, and a huge interest has been devoted to understanding the mechanisms of virulence [1] and environmental-friendly biocontrol approaches [2]. Certain biocontrol strategies rely on the exploitation of beneficial traits of plant growth-promoting (PGP) microorganisms that are naturally associated with plants [3].

Little attention has been dedicated to bacteria associated with grapevine, and, subsequently, relatively few studies are available. It was shown that grapevine tissues, including flowers, berries pulp, and seeds, host an endophytic community that encompasses, among others, bacteria affiliated to the *Bacillus*, *Burkholderia,* and *Pseudomonas* genera [4–9]. Despite these studies, little information is currently available on the taxonomic and functional diversity of the bacterial communities associated with the endosphere and root system of grapevine. Recently, we observed that *Enterobacteriaceae* were dominant in the root system of five different Barbera cv. rootstocks cultivated in Oltrepò Pavese, with a differential genera distribution and colonization of the rhizosphere and endosphere [10].

Even fewer studies have investigated the potential of grapevine-associated bacteria in promoting plant resistance to abiotic stresses [11, 12]. Water deprivation reduces fruit yield remarkably and causes quality losses, and most of the grapevine-growing regions worldwide suffer seasonal periods of drought [13]. Although generally referred to as a drought-resistant plant, grapevine is severely affected in terms of fruit yield and quality by the cooccurrence of elevated temperatures and high evaporation rates that reduce carbohydrate content in berries and cause wilting of leaves. Even at temperate latitudes and cool climates, extensive periods of drought coupled with certain soil types and topography in the vineyard may determine anticipated harvests and influence phenology [14]. In both managed and natural ecosystems, the interaction of plants with the associated native-drought resistant microbiome is an important factor in supporting plant health and physiology under water stress [15, 16]. As sessile organisms, plants adopt different strategies to persist in the face of unfavourable environmental settings, including phenotypic plasticity or “escape and migrate” mechanisms. Rapid adaptation of plants to low soil moisture is achieved through changes in the structure and functionality of the belowground microbiome [17]. The selective force exerted by water scarcity combined with unfavourable harsh environmental conditions in arid lands enriches the rhizosphere of beneficial bacteria that exhibit antagonistic activity against phytopathogens and promote plant resistance to drought [16, 18]. Recently, we observed that the treatment of grape plantlets with selected bacteria determined an increase in epigeal biomass and the formation of a larger root system during drought stress [10].

It is well known that bacteria play key roles in promoting plant growth in conventional and extreme ecosystems [16, 19], and that a plethora of environmental factors such as the cultivar type or pedoclimatic conditions can affect and modulate the structure of bacterial microbiomes [20]. However, the influence of such environmental factors on the PGP potential of root-associated bacteria is poorly understood.

The present study aims to assess the range of bacterial diversity and functional PGP potential of culturable bacteria associated with grapevine roots growing in three different agrosystems in the Mediterranean basin, in order to evaluate whether such PGP potential is independent from the specific environmental conditions. The sampling sites were located in North Italy, North Tunisia, and North Egypt, drawing a latitudinal/aridity macro-transect in the Mediterranean basin. The structure of the bacterial communities associated with the root endosphere and soil of grapevines in the three sites was dissected by 16S rRNA gene-based PCR-DGGE (denaturing gradient gel electrophoresis) analysis. The results were compared with the diversity of the culturable bacteria and their PGP potential.

## 2. Materials and Methods

### 2.1. Study Area and Sample Collection

Three different agroclimatic regions of the Mediterranean basin were chosen for sampling. During July 2008, grapevine root samples were collected from vineyards located in Mornag, North Tunisia, (TN) and in an arid farm 30 km north-west of Cairo, Egypt (ET). In July 2009, grapevine roots were collected from a vineyard of the “Le Frecce” farm, North Italy (IT). The sites were located along a latitude gradient, from 44° to 30°N (IT: 44°57′N, TN: 36°84′N, ET: 30°2′N). The roots (root tissues and rhizosphere) of healthy grapevine plants were collected at 50–60 cm depth. After removing the roots, the root-surrounding soil was collected and the bulk soil was sampled at a distance of 4 m from the grapevine plants. All soil and root samples were collected under sterile conditions using sterile tools. Recovered samples were stored at −20°C for molecular analysis or at 4°C for isolation and processed the following day in the laboratory.

### 2.2. Total DNA Extraction and PCR from All Root System Fractions

Grapevine roots with attached soil particles were placed in a 50 mL screw-cap tube containing 9 mL of physiological solution (9 g/L NaCl) to separate the rhizosphere soil by vortexing. The root tissues removed from the 50 mL screw-cap tube were sterilized as described by Sun et al. [21] by immersion in 70% ethanol for 3 min, sodium hypochlorite solution (2.5% available Cl^−^) for 5 min, and 70% ethanol for 30 seconds. After these treatments, the tissues were washed five times with sterile distilled water. The efficacy of the sterilization method was verified by plating the water of the last washing step on PAF medium (10 g/L proteose peptone, 10 g/L hydrolyzed casein, 3 g/L MgSO_4_, 1.5 g/L K_2_HPO_4_, 10 mL/L glycerol, and 15 g/L agar for solid medium). Total DNA was extracted from the soil fractions (rhizosphere, root-surrounding soil, and bulk soil) and root tissues using a Power Soil kit (MoBio) and DNeasy Plant kit (Qiagen), respectively, according to the manufacturer's procedure. The DNA was quantified and stored at −20°C until use. PCR amplification of the 16S rRNA gene was performed using the 907R and 357F primers, adding a GC-clamp to the forward primer [22]. PCR reaction was performed in 0.2 mL tubes in a final volume of 50 *μ*L containing the 1x diluted buffer, 1.5 mM MgCl_2_, 5% DMSO, 0.12 mM of a mixture of dNTPs, 0.3 *µ*M of each primer, 1 U Taq polymerase, and 10 ng of template. When necessary, DNA was properly diluted. The amplification program consisted of an initial denaturing step at 94°C for 4 min, followed by 10 cycles of 94°C for 0.5 min, 61°C for 1 min, and 72°C for 1 min, followed by further 20 cycles at 94°C for 0.5 min, 56°C for 1 min, 72°C for 1 min, and a final extension at 72°C for 7 min. Two *μ*L of the PCR products was analyzed by electrophoresis in 1% agarose gels.

### 2.3. PCR-DGGE and Profile Analysis

DGGE was performed using polyacrylamide gel (8% of a 37 : 1 acrylamide-bisacrylamide mixture in a Tris acetate EDTA (TAE) 1x buffer, 0.75 mm thick, 16 × 10 cm) with a 45–60% denaturant gradient. Gels were run overnight at 90 V in TAE 1x buffer at 60°C in DCode apparatus (Bio-Rad, Italy). The gels were stained with 1x Sybr Green (Life Technologies) and scanned with gel photo GS-800 system. The DGGE bands were excised from the gels using a sterile scalpel and eluted in 50 *μ*L water at 37°C for 3 hours. The DNA eluted from DGGE bands was amplified using 907R and 357F primers (without the GC-clamp) [22]. The PCR was performed in a final volume of 50 *μ*L with the same conditions as above and using the following protocol: 95°C for 5 min, 30 cycles of 95°C for 1 min, 61°C for 1 min, 72°C for 1 min, and a final extension at 72°C for 7 min. The PCR products obtained were sequenced by Macrogen Inc. (Korea). The DGGE band patterns were converted to a binary dataset by using ImageJ software [23]. Principal component analysis (PCA) was carried out using XLSTAT (version 7.5.2 Addinsoft, France). Analysis of variance along the PCA axis was evaluated using the statistical test ANOVA and Student's *t* test with significance at *P* ≤ 0.05.

The nonparametric statistical test PERMANOVA [24] was used to test the null hypothesis, in which there were no differences between microbial assemblages of sites; PERMANOVA was conducted with the factors of site (fixed, orthogonal, and three levels IT, TN, and ET) and microbial community (fixed, orthogonal, two levels, endophyte, and rhizosphere).

To test for significant relationships among the microbiological assemblage and climate traits [25], a distance-based multivariate analysis for a linear model (DistLM) and distance-based redundancy analysis (dbRDA) [26] were used. PERMANOVA and DistLM were performed with software PERMANOVA + for PRIMER 6 [27].

### 2.4. Isolation of Cultivable Bacteria

One gram of each soil fraction (rhizosphere, root-surrounding soil, and bulk soil) and one gram of triturated root were used as inoculum for ACC-deaminase enrichment culture as described by Penrose and Glick [28]. The medium was supplemented with 100 *μ*g/mL of the fungicide cycloheximide. CFUs per gram of sample were calculated, and 50 colonies from each fraction were randomly selected and propagated on PAF medium plates. One gram of each fraction sample, suspended in 9 mL of sterile physiological solution (9 g/L NaCl), was diluted in 10-fold series and plated in triplicate onto KB medium (20 g/L peptone, 1.5 g/L dipotassium sulphate, 3.2 g/L magnesium dichloride, 10 mL/L glycerol, and pH = 7.2) [29] and on R2A medium (Oxoid). After three days of incubation at 30°C, a total count was performed, and twelve colonies per medium per fraction were randomly selected. For colony purification, all the isolated colony types were spread three times on the original medium. A total of 769 purified isolates were frozen in 25% glycerol at −80°C until use.

### 2.5. DNA Extraction of Isolates, Dereplication and PCR of 16S rRNA Gene

Purified bacterial colonies were resuspended in 50 *μ*L of sterile TE (10 mM Tris/HCl, pH 8, and 1 mM EDTA) in 1.5 mL tubes. Tubes were incubated at 95°C for 8 min and centrifuged at 13000 rpm for 10 min. The supernatant containing the DNA was stored at −20°C and used for PCR. Isolates were dereplicated using the ITS-PCR fingerprinting protocol [30–32]. Two *μ*L of the PCR products was checked by electrophoresis in 1.5% agarose gel and stained with ethidium bromide. Gel images were captured using Gel Doc 2000 system (Bio-Rad, Milan, Italy), and bacteria redundancy was reduced by evaluating the different ITS profiles. One strain per each ITS haplotype was used in the phylogenetic analysis and for further experiments. A total of 331 strains isolated on ACCd [28], R2A (Oxoid), and KB [29] media were characterized by 16S rRNA gene sequencing. The reaction mixture contained the diluted buffer, 1.5 mM of MgCl_2_, 0.12 mM of a mixture of dNTPs, 0.3 *μ*M of each primer, 1 U of Taq polymerase, and 10 ng of template. The universal primers were 27F (3′-AGAGTTTGATCMTGGCTCAG-5′) and 1492R (3′-CTACGGCTACCTTGTTACGA-5′). Conditions for amplification consisted of an initial denaturation at 94°C for 4 min, followed by 35 cycles of 94°C for 0.5 min, 55°C for 1 min, 72°C for 2 min, and a final extension at 72°C for 10 min. The PCR products were checked by electrophoresis in 1% agarose gel, and sequencing service was performed by Macrogen Inc., South Korea.

### 2.6. Characterization of Plant Growth Promoting Activity and Abiotic Stress Resistance

The 331 bacterial strains identified were screened for production of indole acetic acid (IAA), siderophores, exopolysaccharide (EPS) and ammonia productions, mineral phosphate solubilization, protease activity and tolerance to drought, salt, and osmotic stress. The ability of isolated strains to produce IAA was evaluated in the original liquid medium supplemented with L-tryptophan (100 mg/L) as described by Bric et al. [33]. Strains were considered as IAA-producers for concentrations higher than 2 *μ*g/mL. Pure IAA (Sigma-Aldrich Co., Italy) was used to prepare the standard curve and to quantify the amount of IAA produced. Siderophore release was detected in a modified PAF medium (without Fe) using the Chrome Azurol S (CAS) method described by Schwyn and Neilands [34]. Bacterial culture was streaked on a half plate containing growth media. Plates were incubated at 30°C for 7 days, and the formation of orange or pink halos indicated the presence of siderophore. The mineral P-solubilizing ability of the strains was determined on Pikovskaya's liquid medium amended with 0.5% tricalcium phosphate [Ca_3_(PO_4_)_2_] as inorganic P [35]. Exopolysaccharides (EPSs) production was estimated as described by Santarella et al. [36], using the modified Weaver mineral media enriched with 20 g/L sucrose. Bacterial isolates were tested for the production of ammonia in peptone water (peptone 5 g/L). Freshly grown cultures were inoculated in 5 mL peptone water in each tube and incubated for 72 h at 30°C. Nessler's reagent (0.5 mL) was added in each tube. Development of yellow-brownish color indicated NH_3_ production [37]. Proteolytic activity (casein degradation) was determined from clearing zones in skim milk agar after 4 days of incubation at 30°C as described by Nielsen and Sørensen [38]. Resistance to salt was assessed by adding 5, 8, and 10% sodium chloride to culture media and incubating the plate at 30°C for 7 days. The ability to grow at 4, 42, and 50°C was verified in solid media placed in incubators set at the indicated temperatures, and the growth was qualitatively scored after 7 days. Tolerance to osmotic stress was evaluated by adding 10–20% polyethylene glycol (PEG) to the original liquid media.

### 2.7. Nucleotide Sequence Identification and Accession Numbers

Analysis of sequences was performed with the basic sequencing alignment BLAST program run against the database (http://blast.ncbi.nlm.nih.gov/Blast.cgi). The sequences of the partial 16S rRNA genes for isolates were deposited in the GeneBank database under the accession numbers from HF584760 to HF585082, from HE610893 to HE610899, and from HF562892 to HF562897. The DGGE sequences were submitted under the accession numbers from HF678228 to HF678357.

## 3. Results and Discussion

### 3.1. Cultivation-Independent Analysis of Grapevine Root-Associated Bacterial Communities

**Figure 1:**
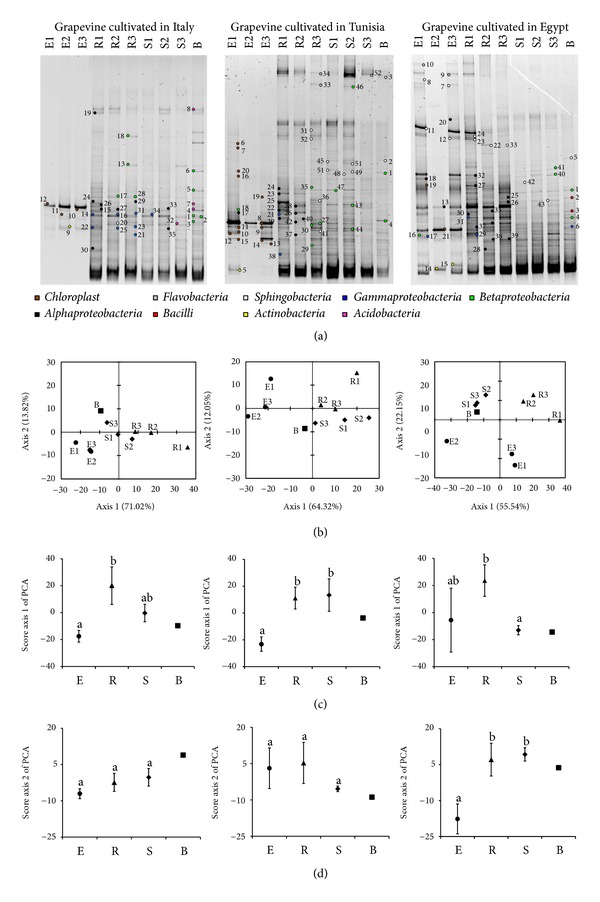
DGGE analysis of grapevine root-associated microbial communities. (a) Representative DGGE gels of the separation of 16S rRNA gene fragments along a denaturing gradient. The analyzed fractions were root tissues (E), rhizosphere (R), root-surrounding soil (S), and bulk soil (B) of three replicate plants of grapevine from a vineyard in Italy (left panel), Tunisia (central panel), and Egypt (right panel). (b) Principal component analysis (PCA) of the plot line profiles that were obtained from DGGE fingerprinting of the bacterial community. E replicates are represented by a circle, R samples by a triangle, S samples by a rhombus, and B by a square. (c)-(d) ANOVA analysis was performed on the average values of the line plot score along axis 1 and 2, respectively, of PCA analysis in order to assess the degree of similarities among plant and soil-associated bacterial communities in the three study sites. Different letters (a and b), shown at the top of the scatter plots in the graph, indicate a statistical significance at *P* ≤ 0.05 according to ANOVA analysis.

The diversity of bacterial communities associated with the grapevine root system from each of the three studied regions was investigated through the analysis of the diversity of the 16S rRNA gene in the root tissues (E), rhizosphere (R), and root-surrounding soil (S) fractions of three replicate plants including bulk soil (B) as a comparison (Figure 1(a)). Multiple-band PCR-DGGE profiles were observed in all fractions (E, R, and S) of the three grapevine root systems and in the respective bulk soils (Figure 1(a)). The endophyte fractions from the soil samples were different, showing the simplest profile composed of a limited number of bands compared to the other root system samples (Figure 1(a)). This observation was confirmed by PCA of the PCR-DGGE band patterns that showed a sharp separation between E-associated bacterial communities and those of soil fractions (Figures 1(b) and 1(c)). The reduced microbial diversity in root tissues may reflect specific physiological requirements to enter the interior of the roots and establish as endophytic populations [39]. The soil fractions showed a multiple-band profile in the DGGE gels, indicating the hosting of a high number of different bacterial taxa. Several shared bands were observed among the R, S, and B fractions, suggesting that similar bacteria may have colonized different soil portions (Figure 1(a)). This was observed in the case of grapevines cultivated in North Italy and Tunisia where R, S, and B fractions tended to cluster together according to the PCA (Figure 1(b)). On the contrary, a clear rhizosphere effect was observed in the root system of grapevines growing in Egypt; the structure of the rhizosphere bacterial communities diverged from the S and B samples (Figure 1(c)), according to the scores of axis 1 of PCA, whereas a statistically significant difference was detected among the bacterial communities associated with the endosphere and soil fractions in Egypt, according to the scores of axis 2 of PCA (Figure 1(d)). Generally, the rhizosphere is defined as a transition zone between the root surface and soil, where the released exudates favour microbial proliferation, inducing changes in the structure and chemical-physical features of the soil [40]. The rhizosphere effect is especially pronounced in nutrient-poor soils and under severe abiotic stresses, as previously observed for herbaceous and arboreal plants grown in arid lands [16, 41].

**Figure 2:**
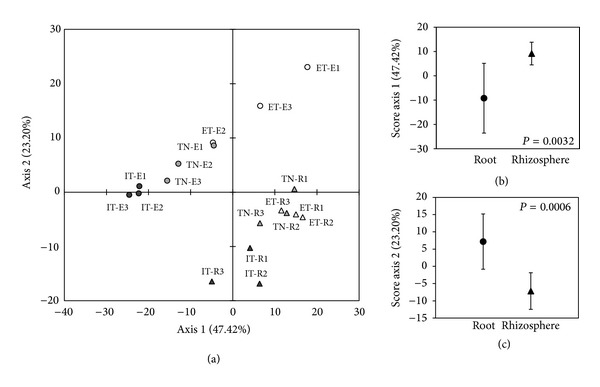
Comparison of the structure of endosphere and rhizosphere bacterial communities associated with grapevines along an aridity transect in the Mediterranean basin. (a) Principal component analysis (PCA) of the PCR-DGGE profiles of endophyte (E) and rhizosphere (R) fractions associated with grapevine cultivated in Italy (IT), Tunisia (TN), and Egypt (ET). Samples were run and analyzed in triplicate. (b)-(c) Statistical analysis was applied to the average values of endophyte and rhizosphere samples along axis 1 and axis 2, respectively, of the PCA analysis. Statistical significance (*P*) was evaluated according to the Student's *t*-test and is indicated in every graph.

We focused on the comparison of the structure of endosphere and rhizosphere-associated microbial communities of grapevines along the investigated transect (Figure 2(a)). A differentiation of the bacterial diversity among the two different fractions was observed by PCA of the PCR-DGGE profiles along axis 1 (*P* = 0.0032) and axis 2 (*P* = 0.0006) (Figures 2(b) and 2(c)). The separation between the rhizospheric and endophytic communities along the transect was confirmed by statistical analysis (PERMANOVA, *F* = 30.36; df = 5; *P* = 0.0001). A latitudinal gradient effect was observed in the distribution of the bacterial community in E and R (Figure 2(a)). Bacterial communities associated with grapevines cultivated in Egypt clustered with those of plants cultivated in Tunisia, while the rhizosphere community of grapevine cultivated in Italy was separated in a different cluster, showing a significant difference from the Egyptian and Tunisian communities along axis 2 (*P* < 0.05). Furthermore, the PERMANOVA analysis performed considering the geographical origin showed a significant statistical difference in the distribution of the bacterial communities, indicating an influence of the site of origin in shaping the microbial community of both fractions (PERMANOVA, *F* = 30.45; df = 5; *P* = 0.0078). As previously reported, aridity seems to be the driving force influencing the structure of both Archea and bacterial communities in bare soils [42].

DISTLM multivariate analysis was performed in order to correlate the differences in the structure of microbial communities in the different agroclimatic sites with abiotic environmental parameters (Table 1). The selection of soil microorganisms by plants is a complex process controlled by several factors, often not easily correlated to geochemical settings [43]. Nevertheless, we observed that among abiotic factors, annual rainfall exerted a statistical significant influence in determining the structure of the root system-associated bacterial community (Table 1). It is possible that a cooccurrence of harsh environmental factors, including a hot and dry climate and reduced rainfall, may reflect the differences among the rhizosphere fractions in the three analysed sites (Table 1).

### 3.2. Phylogenetic Affiliation of DGGE Bands Representative of Grapevine Root-Associated Microbial Diversity

**Figure 3:**
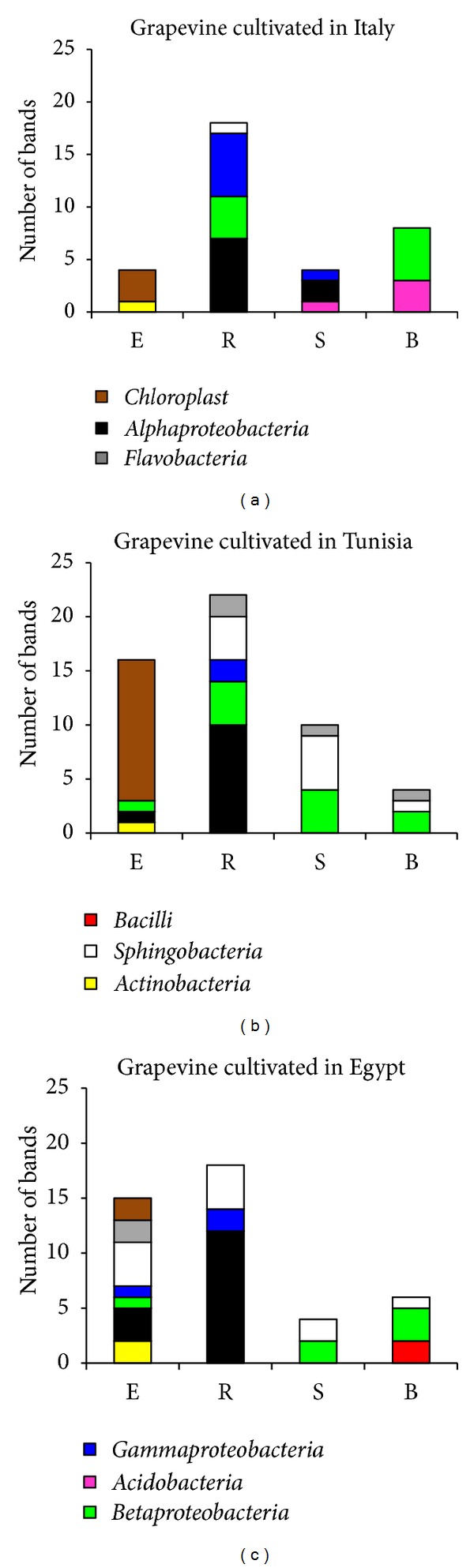
Phylogenetic identification of DNA fragments that were excised from the DGGE gel and successfully amplified and sequenced.

One hundred and twenty-nine bands, representative of all fractions, were excised from the DGGE gel and affiliated to five bacterial phyla by sequencing: *Acidobacteria*, *Actinobacteria*, *Firmicutes, Proteobacteria,* and *Bacteroidetes* (Figures 1(a) and 3, and Table 2). Fifty-one percent of the sequences from E fractions were affiliated to plant chloroplasts, indicating a contamination in DNA extracts with the plastid DNA, as observed in other studies characterizing the endophytic population of grapevine [8, 9] and other plants [21, 44]. Despite plant material contamination, the root tissues from the grapevines grown in Egypt presented a higher bacterial diversity with the *Gammaproteobacteria*, *Sphingobacteria,* and *Flavobacteria* being specific classes of this site. The remaining bands from the endophytic fraction were attributed to *Actinobacteria*, *Alphaproteobacteria,* and *Betaproteobacteria* (Figures 1(a) and 3), which were also dominant in the rhizosphere fractions, confirming that endophytes could represent a subgroup of rhizobacteria which have the ability to enter and establish in the root interior [4, 5, 39]. Although other portals for endophyte entrance into plant tissues cannot be excluded, considering both stomata on the phylloplane of grape flowers and xylem sap [4, 7] or endophyte transmission through seeds [4, 45], root cracks are considered the “hot spot” for bacteria endophytic colonization [37, 46, 47]. Several studies have suggested that the diversity of endophytic bacteria depends on the cultivar and age of the host plant [48], on geochemical and physiological conditions of soil [49] and on climatic variables, such as soil moisture and chemical features [50]. *Alphaproteobacteria* were the best represented class in all rhizospheric soils collected, while *Betaproteobacteria*, which were not detected in Egypt, were equally represented in the other soil fractions in Italy and Tunisia (Figures 1(a) and 3). Despite their diffusion in both reproductive and vegetative plant organs [51], no sequences affiliated to *Burkholderia* spp. were retrieved in our study. Among *Betaproteobacteria*, sequences affiliated to *Massilia* spp. were detected (Table 2). These bacteria were already detected in grape endora during flowering [4] and display a copiotrophic lifestyle at the root niche [52]. Some members affiliated to this genus have been shown to efficiently colonize cucumber seed coats and radicles and are found to be associated with the hyphae of mycorrhizae infecting germinating seeds [52]. Sequences related to *Chryseobacterium* spp. were to be found associated with grape plants in Tunisia and Egypt (Table 2). A *C. balustinum* strain was characterized by its stimulation of the release of flavonoids in *Phaseolus vulgaris*, specifically produced during plant-bacteria interaction, that can presumably be metabolized by the bacterium [53]. *Gammaproteobacteria* sequences were retrieved in rhizosphere samples from the vineyard in Italy, while its relative abundance was lower in grapevine rhizosphere samples from Tunisia (Figure 3). Interestingly, *Rhizobiales *spp. was observed only in R fractions, in agreement with previous findings regarding the isolation of *Rhizobium* spp. from grape rhizosphere during flowering [4]. Their role in supporting plant growth or nutrient assimilation remains to be elucidated, considering that under low soil moisture plants suffer from a disturbed bioavailability of nutrients [17]. Indeed, as a consequence of its ability to release EPS, a *Rhizobium* sp. strain contributed to improve root-adhering soil (RAS) aggregation in sunflower plantlets grown both in drained and dry soils [54]. *Acidobacteria* were revealed only in soil fractions loosely or nonassociated with roots (S and B) in grapevines cultivated in Italy. No bacteria affiliated to the *Firmicutes *phylum were observed in the root system (Figure 3). Members of the *Flavobacteriaceae* class, that were retrieved in Egypt (endosphere) and Tunisia (rhizosphere), were classified among the most abundant bacteria in root and phyllosphere of *Arabidopsis thaliana* plants grown in the wild [55]. A larger genome size has been advocated as a possible reason for the success of *Flavobacterium* spp. in plant organ colonization, supporting a higher metabolic flexibility for the use of complex sugar compounds secreted by plants [56].

Our findings on the grapevine-associated bacterial community structure are in agreement with other studies carried out on grape roots. Few bacterial groups were found in grapevine tissues using a diverse array of methods including 16S rRNA gene libraries, length heterogeneity PCR, and FISH hybridization [4, 9, 57]. Among other bacteria, FISH analysis detected cells affiliated to *Firmicutes* and *Gammaproteobacteria* adhering on epidermal cells, associated with xylem elements and colonizing different organs of flowers, including ovaries [4]. ARDRA profiles demonstrated that flowers, fruits, and seeds host a rather low diversity of endophytes compared to rhizosphere and root tissues, suggesting that specific metabolic skills are required to translocate from the root interior to other plant tissues [4]. Among the genetic determinants affecting the endophyte lifestyle, a gene codifying for *β*-galactosidase and two genes for the expression of acyl homoserine-lactones (AHL) were recently found in the genome of *Methylobacterium* sp. strain GFX4 [58]. This strain, associated with the xylem of Riesling grapevines, could communicate with other xylem-associated endophytes, potentially influencing their behaviours; by partially digesting galactan, this strain could also improve its colonization in the xylem through modification of the plant cell wall [58]. Endophyte communication through the release of AHL has been documented for strains isolated from both grapevine and sugarcane and could play a, still unknown, role for bacteria establishment in plant tissues [59].

### 3.3. Diversity of Culturable Bacteria Associated with Grapevine Root Systems

**Figure 4:**
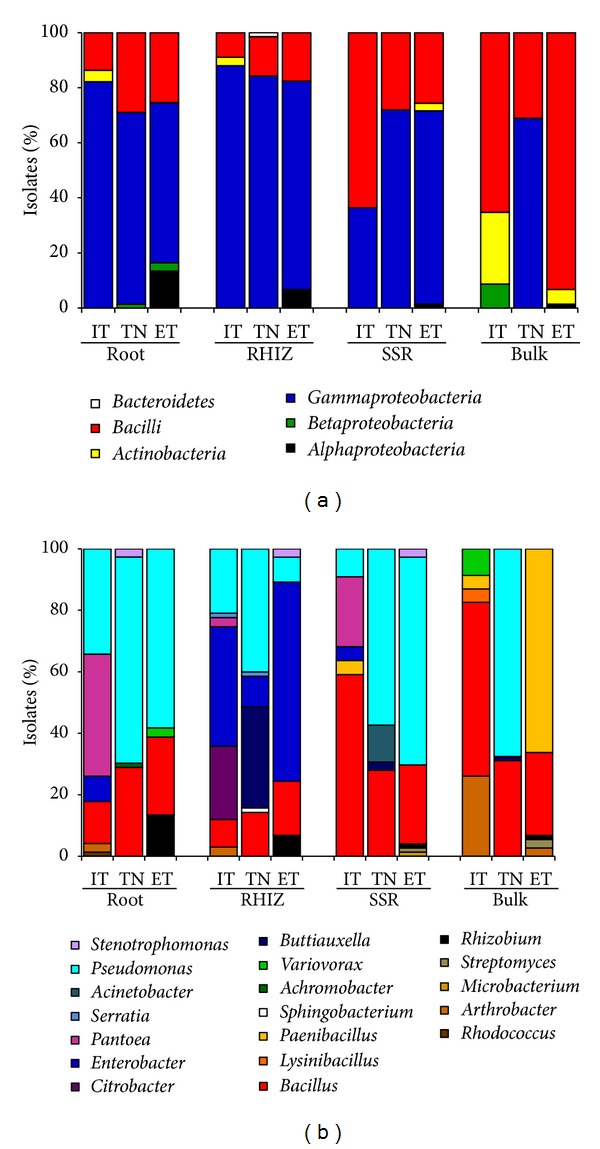
Phylogenetic identification of culturable bacteria associated with grapevine plant and soil fractions. (a)-(b) Bacteria repartition in endosphere (ROOT), rhizosphere (RHIZ), root-surrounding soil (SSR), and bulk soil (BULK) of grapevine grown in Italy (IT), Tunisia (TN), and Egypt (ET) according to the class and genus level, respectively.

The isolation of native bacterial species associated with grapevine cultivated in North Italy, Tunisia, and Egypt was performed using different media in order to select for oligotrophic bacteria, *Pseudomonadaceae,* and ACC deaminating bacteria, already well documented as plant growth promoters [60–62]. The highest number of cultivable bacteria expressed as colony-forming units (CFUs) per gram of sample was in the rhizosphere (10^8^-10^9^) and progressively decreased passing from the S (10^7^–10^9^) to the B fractions (10^5^–10^7^). The root tissues presented the lowest values (10^5^–10^7^), supporting previous data obtained on grapevine-associated bacterial communities [4, 7, 63]. It is noteworthy that bulk soil and endosphere host similar bacterial communities, in terms of size, suggesting that the ability to thrive in plant tissues requires specific genetic requirements [39]. A total of 769 isolates were obtained from the selected agar media as representative of the different populations/morphologies. To reduce genotypic redundancy, the ACCd bacterial collection was dereplicated using ITS-PCR fingerprinting, and a representative bacterium from each haplotype was selected for further identification and characterization. In total, 331 strains were identified by partial sequencing of the 16S rRNA gene. The phylogenetic identification of culturable bacteria highlighted the diversity in terms of composition of the different fractions, revealing a predominance of Gram-negative bacteria (66%), belonging to the *Gammaproteobacteria *(63%), *Alphaproteobacteria *(2%), and *Betaproteobacteria* (1%) subclasses. The remaining Gram-positive isolates were affiliated to the *Firmicutes* (31%), *Actinobacteria* (2%), and *Bacteroidetes* (1%) classes (Figure 4(a)). Members of these taxa have been found to be associated with other grape plants cultivated in Italy [9, 57], France [4, 63], Turkey [64], Nova Scotia [7], and Australia [8]. A high Shannon-Weaver diversity index, calculated from the number of individuals per genus, was found within the rhizosphere of grapes cultivated in Italy (*H*′ = 1.52) and Tunisia (*H*′ = 1.36) and in the endosphere of ungrafted Barbera in Italy (*H*′ = 1.37). On the contrary, the bacterial communities associated with the root system of grapes cultivated in Egypt presented lower diversity values (*H*′ = 1.037 in E and *H*′ = 1.07 in R). The high genetic diversity of grape root systems presumably resulted from the combined effects of root exudates and agricultural management practices, particularly at the lower latitude site where the arid pedoclimatic condition may have influenced the bacterial community composition [43]. The microbial community in the bulk soil, not directly influenced by the root system or agricultural practices, showed the lowest diversity indexes, particularly in the samples collected from the Southern Mediterranean sites (*H*′ = 0.69 in Tunisia and *H*′ = 0.88 in Egypt). On the contrary, the bulk soil collected in vineyards in Italy recorded a higher Shannon index (*H*′ = 1.158), probably because of a more structured soil texture that is able to host a richer microbial community [43]. Significant differences were observed in the structure of the bacterial communities in the analyzed vineyards, in particular for the differential distribution pattern of the major bacterial genera (Figure 4(b)). According to the cluster analysis at the genus level performed on the entire strain collection, the composition of the cultivable communities associated with grapes cultivated in Egypt and Tunisia shared a higher similarity (82%) than those in Italy (68%). A similar profile of bacteria distribution was observed in the root systems of all grapevines studied, with the dominance of *Gammaproteobacteria* followed by* Firmicutes*. Even at the genus level, differences are highlighted in the bacteria distribution in plant and soil fractions and within the studied sites, particularly among Tunisia and Egypt. The rhizosphere of grapes cultivated in these countries differed for the different percentages of bacteria affiliated to *Pseudomonas* (40% in Tunisia and 8% in Egypt) and *Enterobacter* (10% in Tunisia and 65% in Egypt). Endophytes from the Italian plants were dominated by *Pantoea* (40%), a genus that was not detected in grapes grown at lower latitudes, followed by *Pseudomonas* (34%), *Bacillus* (14%), *Enterobacter* (8%), *Arthrobacter* (3%), and *Rhodococcus* (1%). The genus *Pantoea* has been frequently associated with grape tissues and may contribute to prime plants for accelerated phytoalexin production after *B. cinerea* challenge [65]; its plant growth potential has already been documented for several model plants [66, 67]. Egypt and Tunisia grape root tissues hosted a higher percentage of isolates affiliated to *Pseudomonas* and *Bacillus* genera, confirming previous findings on bacteria community composition in grape tissues as assessed through isolation and culture-independent methods [4, 8]. *Pseudomonas* spp. in particular was abundant in the soil fractions from Tunisia (55%), although it was also observed in the other two sites (25% in Egypt and 14% in Italy), confirming the widespread diffusion of this genus in root-influenced soils [68]. Despite the presence of sequences affiliated to *Rhizobiales* in all three vineyards, as observed by DGGE analysis, isolates belonging to the *Rhizobium* genus were retrieved only in the root tissues of grapes cultivated in Egypt (13%). The role of these bacteria remains to be elucidated, although their association with other crops has been proposed for field applications [69]. The most abundant genera that were associated with the endosphere were also retrieved from the rhizosphere, although at lower percentages. This observation strongly supports the theory regarding endophytic bacteria entry from the root system to spread in plant tissues through xylem translocation, as previously documented for both beneficial and pathogenic strains such as *Agrobacterium tumefaciens* [70], *Burkholderia* sp. strain PsJN [5], *Yersinia enterocolitica* strain [71], and *Xylella fastidiosa *[72].

Isolates from the R fraction were mainly affiliated to the *Enterobacteriaceae* family. *Enterobacter* that was shared among the rhizosphere soils of the three sites was found at a higher percentage in the soils in Egypt (65%) and Italy (39%). In the rhizosphere of grapes from Tunisia, *Buttiauxiella* (33%) was the main genus of the *Enterobacteriaceae*, while in Italy *Citrobacter* accounted for 24% of the local collection (Figure 3(b)). The predominance of bacteria affiliated to the *Enterobacteriaceae* family could be attributed to the application of crop management techniques based on the use of natural fertilizers such as manure and plant residues [16, 18]. Nevertheless, representative species of *Enterobacteriaceae* were widespread in several plant systems, suggesting a role in plant colonization and plant promotion also during stressful conditions [73]. In the S fractions, less influenced by root exudates, the isolation frequency of *Bacillus* increased. This shift in the bacterial community composition was evident in the bulk soil, not subjected to amendment and irrigation processes. In both S and B fractions, the *Enterobacteriaceae* disappeared, while the spore-forming bacteria (*Firmicutes* and *Actinobacteria*), typical of poorly structured soil, increased in incidence reaching 98% in the bulk soil in Egypt, 91% in Italy, and 31% in Tunisia (Figure 3(b)). The prevailing genera* Arthrobacter*, *Bacillus,* and *Paenibacillus* can survive as resting cells or spores under adverse environmental conditions, hence, making them typical taxa of uncultivated and arid soils [43].

The observed diversity of the culturable fraction is in agreement with the findings provided by PCR-DGGE analysis. Indeed, the same microbial taxa were retrieved with the exception of *Acidobacteria* that were detected only through molecular analysis. Despite the fact that the cultivation approach generally favours some taxa [74] and that the PCR-based techniques of metagenomes are biased by the preferential amplification of certain bacterial groups [75], the combination of cultivation independent and dependent techniques revealed sharp differences among the structure of microbial communities associated with root systems of grapes cultivated in three Mediterranean regions.

### 3.4. Determining the PGP Potential of Grapevine-Associated Bacteria

**Figure 5:**
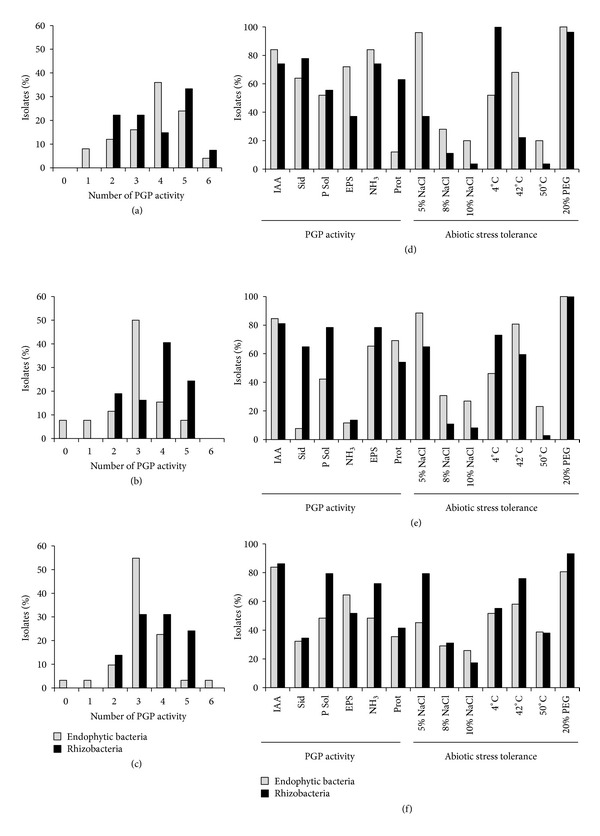
PGP potential of grapevine-associated bacteria. (a)–(c) Percentage of isolates showing an increasing number of PGP abilities in the strain collection isolated from grapevine grown in vineyards located in Italy, Tunisia, and Egypt, respectively. (d)–(f) Percentage of isolates displaying the assayed PGP traits and abiotic stress tolerance in the bacterial collection of strains associated with grapevine cultivated in Italy, Tunisia, and Egypt, respectively.

One hundred and seventy-five isolates of the de-replicated collection, 93 from the rhizosphere and 82 endophytes, were further screened *in vitro* for the presence of PGP traits. To characterize their PGP potential, auxin (IAA) production, phosphate solubilization, ammonia and siderophores productions, protease activity, and exopolysaccharide (EPS) release were evaluated. The majority (95%) of isolates showed multiple PGP activities, which may promote plant growth directly, indirectly, or synergistically. In particular, none of the rhizobacteria and only 4% of the endophytes showed only one or no activity, while about 80% of isolates from both fractions displayed more than three PGP activities. Interestingly, the distribution of the number of PGP activities displayed by the strains in the three study sites revealed a similar distribution profile (96% in Italy, 97% in Tunisia, and 94% in Egypt), supporting the hypothesis that a huge functional PGP potential is maintained in grapevine root systems (Figures 5(a), 5(b), and 5(c)). Among the most common PGP abilities was the production of auxin-like compounds (82%), followed by the synthesis of ammonia (70%) and the solubilization of insoluble phosphates (61%). In terms of auxins, IAA role in the stimulation and elongation of the root apparatus is well documented, extending the root surface involved in nutrient and water uptake [76]. In our bacterial collection, the IAA production was equally distributed among endophytic (84%) and rhizospheric (80%) bacteria, and a similar trend was observed in all three sites along the latitude transect (79% in Italy, 82% in Tunisia, and 85% in Egypt) (Figures 5(d), 5(e), and 5(f)), in agreement with previous observations that IAA synthesis is a widespread PGP trait [77]. The IAA production ranged from 2.11 to 36.2 *μ*gmL^−1^, with the highest amount produced by the isolates from the rhizosphere and endosphere of grape cultivated in Italy (14.4 and 18.6 *μ*gmL^−1^ in E and R fractions, resp.). The production of ammonia can indirectly influence plant growth through the supply of nitrogen [78]. In the present investigation, 70% of isolates displayed ammonia production. A similar distribution among the endophytic and rhizospheric bacteria was observed (Figures 5(d), 5(e), and 5(f)), with the exception of strains isolated from Italian root tissues (48%). Similarly, phosphate solubilisation ability was exhibited by 61% of the isolates collected. Phosphorous is a key nutrient for plant growth, representing one of the main factors limiting plant development and productivity [79]. The ability of rhizobacteria to solubilize phosphate (79% in Egypt, 78% in Tunisia, and 56% in Italy) through the production of organic acids or phytases can support plant growth in nutrient-poor soils in drought-prone ecosystems, such as those studied in this work [80–82]. Moreover, the isolates showed protease (46%), siderophore production (47%), and EPS release (41%). The synthesis of protease presented a similar pattern of distribution (about 50%) along all the fractions of grape root system analyzed, except for the endophytes associated with grapevine from Italy (12%). Several siderophore-producing bacteria were observed mainly in the rhizosphere (78% in Italy, 65% in Tunisia, and 35% in Egypt), probably because this PGP trait confers competitive colonization ability in iron-limiting soil and exerts a biocontrol role, reducing iron-dependent spore germination of fungi. A high percentage of siderophore-releasing bacteria was recorded only among the endophytic bacteria isolated from grapes cultivated in Italy (64%). Finally, EPS production was qualitatively evaluated. Only 49% of endophyte and 32% of rhizobacteria were able to produce EPS, with the highest percentages observed for the isolates associated with grape roots from Egypt (65% in R and 52% in E). Bacteria adapted to arid environments are well known to protect themselves from extreme climate conditions, producing EPS-rich biofilms that entrap water molecules and thus retaining moisture [83]. Bacterial EPS production in clay-rich soils, such as those in Italy, may presumably play an additional role for favoring root penetration in hard soils such as dry clay soils [84].

Further analyses were performed to evaluate bacteria resistance to abiotic stresses that are often associated with drought, such as increased salinization of soils and air temperatures that rise up to 50°C in daytime and drop down during the night. Thus, we analyzed the ability of bacteria to survive in the presence of increasing concentrations of salt, to grow despite temperature fluctuations (4, 42 and 50°C), and to thrive in conditions of low water availability (Figures 5(d), 5(e), and 5(f)). Salinization of dry soils, together with drought and temperature variations, deeply hamper plant physiology and development [85]. As expected, the number of strains resistant to salt decreased with increasing NaCl concentration (5%, 8%, and 10%). While 67% of the isolates were able to grow on media containing 5% NaCl, this percentage decreased to 22% and 16% at 8% and 10% NaCl, respectively. As shown in Figure 4, even at lower NaCl concentrations, bacteria from the endophytic fraction showed sensitivity to salt, particularly among bacteria isolated from Egyptian grape root, where 55% of isolates could not grow in the presence of salt. On the contrary, rhizobacteria isolated from Egypt included the highest proportion of isolates resistant to salinity (79%), followed by those isolated from Tunisia (64%) and Italy (37%). At increasing concentrations of salt, the percentage of resistant isolates decreased, with only 17% of isolates able to grow at 10% NaCl. In particular, the capacity to tolerate high salt concentration followed the latitudinal/aridity transect from the south to the north (Figures 5(a), 5(b), and 5(c)), with percentages ranging from 31% in the rhizosphere of grapes from Egypt to 4% in the rhizosphere from Italy. Although halotolerance has been studied in bacteria affiliated to *Halomonas* spp. [86], this study highlights that even under soil dryness bacteria with moderate halotolerance can be observed. Interestingly, almost all of the isolates were able to grow under low water availability induced by PEG [87]. All the strains isolated in Tunisia and 98% of those associated with Italian grapevine root systems (100% in E and 96% in R) were able to grow at 20% of PEG, while slightly lower percentages of tolerance were observed for bacteria isolated from grapes cultivated in Egypt, that is, 80% in the endosphere and 93% in the rhizosphere (Figures 5(d), 5(e), and 5(f)). During drought, belowground microbiomes survive by using water reserves in soils that, in turn, are rapidly depleted by earlier development of plants [88]. Thus, osmotic tolerance is a key feature for microbial survival.

Finally, we observed that 63% and 61% of the isolates could grow at 4°C and 42°C, respectively. The majority (52% in E and 100% in R) of the strains isolated from Italian vineyards were able to grow at 4°C (Figures 5(d), 5(e), and 5(f)), presumably adapted to the cold temperatures in autumn and winter when the average air temperatures can be as low as −1°C [25]. On the contrary, only bacteria associated with grapevines grown in Egypt presented resistance to high temperatures, with 39% and 38% of strains isolated from root and rhizosphere, respectively, capable of growing at 50°C, probably being adapted to hot summer temperatures [89]. Global warming is predicted to affect microbial communities, hampering their physiology and growth [90]. The 29% of the collected strains were able to grow both at low and high temperatures, confirming that these isolates were adapted to the peculiar temperature fluctuations of the studied environments. The 23% of isolates presented the potential to express their PGP ability in unfavourable environmental conditions influenced by drought, simultaneously showing halotolerance, resistance to a variable temperature range, and low water availability.

## 4. Conclusions

The Mediterranean is a closed basin, encompassing subtropical, arid, and continental climates that, to date, are rapidly changing through the increase in length and extent of dry periods [91]. Among the most cultivated and economically relevant crops, *Vitis vinifera* is widespread at all the basin latitudes, and grape quality and yields are affected by prolonged drought events. The findings reported in the present study contribute to expand the knowledge on the diversity and PGP potential of grapevine-associated bacteria under three different agroclimatic conditions. Culture-dependent and independent techniques highlighted that, according to a specific and yet undefined selection mediated by the different cultivars and rainfall and temperature regimes, the rhizosphere and endosphere microbial communities are different among the three different sites. Indeed, many environmental factors may explain such diversity, such as soil moisture and temperature [79]. While summer temperatures in Italy, Tunisia, and Egypt are quite high and of a similar magnitude, in winter, grapevines in the different study sites experience different levels of low and freezing temperatures that presumably contribute to the modelling of the grape-associated microbial communities. Despite the different biopedoclimatic conditions of the three studied sites (in terms of different cultivars, soil types, and climate conditions), a large set of PGP abilities is still displayed by the respective collections of isolates, independently from the site of origin, and with a similar profile in terms of the number of PGP traits and activities. A redundant functional capability of the isolates from the root systems has been recorded in all the three sites, indicating that in these three environments, the root bacterial communities are adapted to the respective conditions. For instance, insoluble phosphate solubilisation, auxin synthesis, and ammonia production were exhibited by multiple strains in all three sites, highlighting that despite site-specific chemical settings, grapevines in the three investigated agroclimatic regions share similar physiological requirements that are, at least partially, provided by PGP-associated bacteria. Such results suggest that the great diversity of the bacterial world has enough resources to provide functional redundancy in rather different environments, such as those examined, and that a functional homeostasis of the root system bacterial communities may sustain grapevine life in rather different environmental settings.

## Figures and Tables

**Table 1:** Relationships between endophytic/rhizosphere bacterial assemblages and climate features of different regions using nonparametric multivariate multiple regression analysis (DISTLM). Partial (conditional) test where the amount explained by each variable added to the model is conditional on variables already in the model.

Variable	IT	TN	ET	AIC	*F*	*P*	% Var	% Cumul	Res. df
Annual rainfall (mm)	809.1	561.1	39.7	110.09	27.749	0.0048	28.388	28.388	7
Summer rainfall (mm)	101.4	7.4	0.1	109.66	18.639	>0.05	16.973	45.362	6
Average rainfall (mm)	9	1	0	109.66	0	>0.05	<0.0001	45.362	6
Max temperature (°C)	31	30	35	109.66	0	>0.05	<0.0001	45.362	6
Min temperature (°C)	19	21	23	109.66	0	>0.05	<0.0001	45.362	6
Average temperature (°C)	25	25.5	29	109.66	0	>0.05	<0.0001	45.362	6
Latitude	44.6	36.8	30.2	109.66	0	>0.05	<0.0001	45.362	6
Longitude	9.1	10.1	31.2	109.66	0	>0.05	<0.0001	45.362	6
Irradiation (w/m^2^)	720	870	880	109.66	0	>0.05	<0.0001	45.362	6

AIC: coefficient of regression; *F*: value of pseudo *F*; *P*: significance of *F*; % Var: percentage of variance explained by each single variable; % Cumul: cumulative percentage of variance explained; Res. df: residual degrees of freedom.

**Table 2:** Identification of the dominant bands in the PCR-DGGE fingerprinting profiles (marked in Figure 1). The codes of the different fractions of the grapevine root systems are as follows: E, Endosphere; R, rhizosphere; S, root-surrounding soil; B, bulk soil.

Frac.	DGGE band	Closest relative (NCBI database)	Acc. N°	%	Closest describe relative (Ez Taxon database)	Acc. N°	%	Class
B	IT-01	*Ramlibacter* sp.	AY429716	88	*Curvibacter fontanus *	AB120963	87	*Betaproteobacteria *
B	IT-02	Unc. *Comamonadaceae *	AY360707	86	*Curvibacter fontanus *	AB120963	85	*Betaproteobacteria *
B	IT-03	Unc. *Variovorax* sp.	JN590646	90	*Xylophilus ampelinus *	AF078758	89	*Betaproteobacteria *
B	IT-04	Unc. *Acidobacteria *	EF651005	95	*Desulfomonile limimaris *	AF230531	81	*Acidobacteria *
B	IT-05	Unc. bacterium	HQ393158	98	*Massilia plicata *	AY966000	96	*Betaproteobacteria *
B	IT-06	*Massilia* sp.	JF279920	99	*Massilia niabensis *	EU808006	99	*Betaproteobacteria *
B	IT-07	Unc. bacterium	AY274152	97	*Solibacter usitatus *	CP000473	92	*Acidobacteria *
B	IT-08	Unc. *Acidobacteria *	GU257870	94	*Chloracidobacterium thermophilum *	CP002514	80	*Acidobacteria *
E	IT-09	*Micromonospora* sp.	FR692086	92	*Micromonospora peucetia *	X92603	90	*Actinobacteria *
E	IT-10	Chloroplast	U189132	99	Chloroplast	AM711640	99	*Eukarya *
E	IT-11	Chloroplast	HQ325745	100	Chloroplast	DQ386163	99	*Eukarya *
E	IT-12	Chloroplast	HQ325745	100	Chloroplast	DQ386163	100	*Eukarya *
R	IT-13	Unc. *Rhodocyclaceae *	EF643420	97	*Thiobacter subterraneus *	AB180657	91	*Betaproteobacteria *
R	IT-14	Unc. bacterium	FR853277	91	*Steroidobacter denitrificans *	EF605262	89	*Gammaproteobacteria *
R	IT-15	Unc. bacterium	JN855310	98	*Bradyrhizobium pachyrhizi *	AY624135	97	*Alphaproteobacteria *
R	IT-16	Unc. *Pseudomonadales *	FJ889292	97	*Steroidobacter denitrificans *	EF605262	96	*Gammaproteobacteria *
R	IT-17	Unc. bacterium	HM445266	92	*Rubrivivax gelatinosus *	D16213	90	*Betaproteobacteria *
R	IT-18	Unc. bacterium	EU881322	99	*Thiobacter subterraneus *	AB180657	90	*Betaproteobacteria *
R	IT-19	Unc. *Rhizobium* sp.	EF074979	92	*Rhizobium giardinii *	U86344	91	*Alphaproteobacteria *
R	IT-20	*Sphingobacterium* sp.	EU580525	97	*Dyadobacter hamtensis *	AJ619978	93	*Sphingobacteria *
R	IT-21	Unc. bacterium	EF019453	86	*Methylogaea oryzae *	EU672873	79	*Gammaproteobacteria *
R	IT-22	Unc. bacterium	EF392989	96	*Steroidobacter denitrificans *	EF605262	87	*Gammaproteobacteria *
R	IT-23	Unc. bacterium	FR853277	95	*Steroidobacter denitrificans *	EF605262	93	*Gammaproteobacteria *
R	IT-24	Unc. bacterium	GU568879	87	*Novosphingobium resinovorum *	EF029110	82	*Alphaproteobacteria *
R	IT-25	Unc. bacterium	FJ479326	93	*Steroidobacter denitrificans *	EF605262	91	*Gammaproteobacteria *
R	IT-26	Unc. bacterium	JN855310	99	*Bradyrhizobium pachyrhizi *	AY624135	98	*Alphaproteobacteria *
R	IT-27	Unc. bacterium	JN855310	95	*Nitrobacter hamburgensis *	CP000319	94	*Alphaproteobacteria *
R	IT-28	Unc. bacterium	DQ643675	93	*Rubrivivax gelatinosus *	D16213	91	*Betaproteobacteria *
R	IT-29	Unc. bacterium	GU291531	94	*Novosphingobium resinovorum *	EF029110	94	*Alphaproteobacteria *
R	IT-30	Unc. *Alphaproteobacteria *	JN371328	81	*Blastochloris sulfoviridis *	D86514	78	*Alphaproteobacteria *
R	IT-31	Unc. *Steroidobacter *	FN297970	100	*Steroidobacter denitrificans *	EF605262	98	*Gammaproteobacteria *
S	IT-32	Unc. *Acidobacteria *	HQ597613	97	*Chloracidobacterium thermophilum *	CP002514	81	*Acidobacteria *
S	IT-33	Unc. bacterium	JN855310	91	*Nitrobacter winogradskyi *	CP000115	90	*Alphaproteobacteria *
S	IT-34	Unc. bacterium	FJ479326	99	*Steroidobacter denitrificans *	EF605262	97	*Gammaproteobacteria *
S	IT-35	Unc. *Alphaproteobacteria *	FJ568851	95	*Donghia mobilis *	FJ455532	91	*Alphaproteobacteria *

B	TN-01	Unc. bacterium	KC541101	100	*Massilia aurea *	AM231588	99	*Betaproteobacteria *
B	TN-02	Unc. bacterium	HM186197	99	*Ohtaekwangia koreensis *	GU117702	93	*Sphingobacteria *
B	TN-03	*Chryseobacterium indoltheticum *	AY468448	99	*Chryseobacterium indoltheticum *	AY468448	98	*Flavobacteria *
B	TN-04	Unc. bacterium	HF546519	83	*Xenophilus azovorans *	AF285414	77	*Betaproteobacteria *
E	TN-05	Unc. bacterium	FN667504	97	*Streptomyces sodiiphilus *	AY236339	96	*Actinobacteria *
E	TN-06	Chloroplast	HQ336404	99	Chloroplast	DQ386163	99	*Cyanobacteria *
E	TN-07	Chloroplast	HQ336404	99	Chloroplast	DQ386163	99	*Cyanobacteria *
E	TN-08	Chloroplast	HQ336404	100	Chloroplast	DQ386163	99	*Cyanobacteria *
E	TN-09	Chloroplast	EU189132	99	Chloroplast	AM711640	99	*Cyanobacteria *
E	TN-10	Chloroplast	HQ336404	99	Chloroplast	DQ386163	99	*Cyanobacteria *
E	TN-11	Chloroplast	HQ336404	100	Chloroplast	DQ386163	92	*Cyanobacteria *
E	TN-12	Chloroplast	HQ336404	99	Chloroplast	DQ386163	99	*Cyanobacteria *
E	TN-13	Chloroplast	EU118126	100	Chloroplast	EU118126	99	*Cyanobacteria *
E	TN-14	Chloroplast	EU118126	100	Chloroplast	EU118126	99	*Cyanobacteria *
E	TN-15	Chloroplast	EU118126	100	Chloroplast	EU118126	100	*Cyanobacteria *
E	TN-16	Chloroplast	EU118126	99	Chloroplast	EU118126	98	*Eukarya *
E	TN-17	*Rhizobium radiobacter *	AJ389904	100	*Rhizobium radiobacter *	AJ389904	100	*Alphaproteobacteria *
E	TN-18	*Hydrogenophilus thermoluteolus *	AB680730	98	*Hydrogenophilus hirschii *	FR749905	98	*Betaproteobacteria *
E	TN-19	Chloroplast	HQ325745	99	Chloroplast	L37580	96	*Eukarya *
E	TN-20	Chloroplast	HQ325745	99	Chloroplast	DQ386163	99	*Eukarya *
R	TN-21	Unc. bacterium	JF198689	89	*Steroidobacter denitrificans *	EF605262	84	*Gammaproteobacteria *
R	TN-22	*Rhizobium sullae *	NR_029330	89	*Rhizobium sullae *	Y10170	82	*Alphaproteobacteria *
R	TN-23	*Rhizobium giardinii *	JX869993	91	*Rhizobium selenitireducens *	EF440185	74	*Alphaproteobacteria *
R	TN-24	*Rhizobium huautlense *	HQ538618	99	*Rhizobium huautlense *	AF025852	99	*Alphaproteobacteria *
R	TN-25	Unc. bacterium	HM328693	99	*Novosphingobium resinovorum *	EF029110	95	*Alphaproteobacteria *
R	TN-26	*Rhizobium etli *	NR_029184	100	*Rhizobium vallis *	FJ839677	99	*Alphaproteobacteria *
R	TN-27	Unc. *Betaproteobacteria *	EU266802	92	*Hydrogenophaga palleronii *	AF019073	78	*Betaproteobacteria *
R	TN-28	*Rhizobium giardinii *	EU410948	99	*Rhizobium endophyticum *	EU867317	98	*Alphaproteobacteria *
R	TN-29	Unc. bacterium	HF546519	85	*Piscinibacter aquaticus *	DQ664244	81	*Betaproteobacteria *
R	TN-30	Unc. bacterium	HE586741	85	*Hydrogenophaga palleronii *	AF019073	78	*Betaproteobacteria *
R	TN-31	Unc. bacterium	JF175892	93	*Niastella populi *	EU877262	81	*Sphingobacteria *
R	TN-32	Unc. bacterium	JF175892	93	*Chitinophaga arvensicola *	D12657	84	*Sphingobacteria *
R	TN-33	Unc. *Flavobacterium* sp.	EU017400	99	*Flavobacterium reichenbachii *	AM177616	84	*Flavobacteria *
R	TN-34	*Flavobacterium* sp.	EF601822	99	*Flavobacterium pectinovorum *	AM230490	98	*Flavobacteria *
R	TN-35	Unc. *Betaproteobacteria *	EF662768	97	*Azoarcus buckelii *	AJ315676	88	*Betaproteobacteria *
R	TN-36	*Sphingobacterium* sp.	EU580525	96	*Dyadobacter hamtensis *	AJ619978	94	*Sphingobacteria *
R	TN-37	*Reichenowi ornate *	AY316684	82	*Aurantimonas altamirensis *	DQ372921	80	*Alphaproteobacteria *
R	TN-38	Unc. *Gammaproteobacteria *	JN648252	97	*Steroidobacter denitrificans *	EF605262	88	*Gammaproteobacteria *
R	TN-39	*Rhizobium* sp.	FN546874	98	*Rhizobium endophyticum *	EU867317	97	*Alphaproteobacteria *
R	TN-40	*Rhizobium* sp.	AM922181	99	*Rhizobium endophyticum *	EU867317	98	*Alphaproteobacteria *
R	TN-41	Unc. bacterium	FJ719038	97	*Dyadobacter hamtensis *	AJ619978	95	*Sphingobacteria *
R	TN-42	*Rhizobium leguminosarum *	HQ853453	99	*Rhizobium vallis *	FJ839677	98	*Alphaproteobacteria *
S	TN-43	Unc. bacterium	AF423222	99	*Thiobacter subterraneus *	AB180657	89	*Betaproteobacteria *
S	TN-44	Unc. *Comamonadaceae *	AY360707	99	*Acidovorax konjaci *	AF078760	97	*Betaproteobacteria *
S	TN-45	Unc. *Sphingobacteriales *	AM934931	99	*Ohtaekwangia koreensis *	GU117702	93	*Sphingobacteria *
S	TN-46	Unc. bacterium	GQ023702	91	*Hydrogenophilus hirschii *	FR749905	91	*Betaproteobacteria *
S	TN-47	Unc. *Burkholderiaceae *	AM935484	99	*Thiobacter subterraneus *	AB180657	90	*Betaproteobacteria *
S	TN-48	*Pedobacter* sp.	AY599662	85	*Pedobacter metabolipauper *	AM491370	84	*Sphingobacteria *
S	TN-49	*Pedobacter africanus *	NR_028977	99	*Pedobacter africanus *	AJ438171	98	*Sphingobacteria *
S	TN-50	Unc. bacterium	HM049699	88	*Pedobacter africanus *	AJ438171	87	*Sphingobacteria *
S	TN-51	Unc. bacterium	HM049699	99	*Pedobacter steynii *	AM491372	98	*Sphingobacteria *
S	TN-52	*Flavobacterium* sp.	HM149210	99	*Flavobacterium pectinovorum *	AM230490	98	*Flavobacteria *

B	ET-01	Unc. *Betaproteobacteria *	JF806989	99	*Thiobacter subterraneus *	AB180657	90	*Betaproteobacteria *
B	ET-02	*Bacillus* sp.	FM992819	99	*Bacillus pocheonensis *	AB245377	98	*Bacilli *
B	ET-03	*Bacillus* sp.	FM992819	87	*Bacillus alcalophilus *	X60603	85	*Bacilli *
B	ET-04	Unc. bacterium	DQ398884	86	*Hydrogenophaga flava *	AF078771	84	*Betaproteobacteria *
B	ET-05	Unc. *Sphingobacteriales *	AM934931	99	*Ohtaekwangia koreensis *	GU117702	92	*Sphingobacteria *
B	ET-06	Unc. bacteria	JF681924	90	*Sterolibacterium denitrificans *	AJ306683	84	*Gammaproteobacteria *
E	ET-07	Unc. bacterium	JQ357881	99	*Chitinophaga pinensis *	CP001699	98	*Sphingobacteria *
E	ET-08	Unc. bacterium	JQ358300	99	*Chitinophaga sancti *	AB078066	98	*Sphingobacteria *
E	ET-09	*Chryseobacterium wanjuense *	AB682410	100	*Chryseobacterium wanjuense *	DQ256729	100	*Flavobacteria *
E	ET-10	*Flavobacterium* sp.	JF915324	99	*Flavobacterium subsaxonicum *	AM934666	96	*Flavobacteria *
E	ET-11	*Chitinophaga* sp.	JQ659659	98	*Chitinophaga ginsengisoli *	AB245374	96	*Sphingobacteria *
E	ET-12	*Chitinophaga* sp.	JQ659659	98	*Chitinophaga ginsengisoli *	AB245374	96	*Sphingobacteria *
E	ET-13	*Novosphingobium* sp.	EU984513	99	*Novosphingobium resinovorum *	EF029110	99	*Alphaproteobacteria *
E	ET-14	Unc. bacterium	FN667504	97	*Streptomyces sodiiphilus *	AY236339	96	*Actinobacteria *
E	ET-15	*Glycomyces scopariae *	JQ342894	99	*Glycomyces scopariae *	EU200682	99	*Actinobacteria *
E	ET-16	*Variovorax paradoxus *	AB680784	99	*Variovorax paradoxus *	D88006	99	*Betaproteobacteria *
E	ET-17	*Pseudoxanthomonas* sp.	JF703645	99	*Pseudoxanthomonas *	AB008507	98	*Gammaproteobacteria *
E	ET-18	*Rhizobium* sp.	AM922181	99	*Rhizobium radiobacter *	AJ389904	99	*Alphaproteobacteria *
E	ET-19	Chloroplast	HQ325745	99	Chloroplast	DQ386163	99	*Eukarya *
E	ET-20	*Rhizobium* sp.	AM922181	85	*Rhizobium radiobacter *	AJ389904	83	*Alphaproteobacteria *
E	ET-21	Chloroplast	HQ325745	100	Chloroplast	DQ386163	99	*Eukarya *
R	ET-22	*Chitinophaga terrae *	AB267724	98	*Chitinophaga niabensis *	EU714259	95	*Sphingobacteria *
R	ET-23	*Chitinophaga* sp.	GQ281772	99	*Chitinophaga niabensis *	EU714259	97	*Sphingobacteria *
R	ET-24	*Chitinophaga* sp.	JQ659659	97	*Chitinophaga sancti *	AB078066	96	*Sphingobacteria *
R	ET-25	*Novosphingobium* sp.	AB453877	90	*Novosphingobium resinovorum *	EF029110	88	*Alphaproteobacteria *
R	ET-26	Unc. bacterium	GQ074926	99	*Novosphingobium resinovorum *	EF029110	97	*Alphaproteobacteria *
R	ET-27	Unc. bacterium	GQ169020	90	*Blastochloris sulfoviridis *	D86514	88	*Alphaproteobacteria *
R	ET-28	Unc. bacterium	DQ814032	99	*Mesorhizobium thiogangeticum *	AJ864462	99	*Alphaproteobacteria *
R	ET-29	*Pseudoxanthomonas mexicana *	JQ660737	100	*Pseudoxanthomonas japonensis *	AB008507	99	*Gammaproteobacteria *
R	ET-30	*Mesorhizobium* sp.	JN688938	94	*Sinorhizobium americanum *	AF506513	93	*Alphaproteobacteria *
R	ET-31	Unc. bacterium	AB540382	82	*Steroidobacter denitrificans *	EF605262	79	*Gammaproteobacteria *
R	ET-32	*Sphingopyxis chilensis *	JF459975	98	*Sphingopyxis panaciterrae *	AB245353	98	*Alphaproteobacteria *
R	ET-33	*Chitinophaga* sp.	JN680879	89	*Chitinophaga niabensis *	EU714259	79	*Sphingobacteria *
R	ET-34	Unc. bacterium	GU291531	100	*Novosphingobium resinovorum *	EF029110	99	*Alphaproteobacteria *
R	ET-35	*Devosia chinhatensis *	EF433462	99	*Devosia chinhatensis *	EF433462	99	*Alphaproteobacteria *
R	ET-36	*Ensifer* sp.	JF450188	99	*Ensifer adhaerens *	AM181733	98	*Alphaproteobacteria *
R	ET-37	*Sinorhizobium fredii *	HQ836172	98	*Sinorhizobium americanum *	AF506513	97	*Alphaproteobacteria *
R	ET-38	*Mesorhizobium loti *	HQ424911	93	*Mesorhizobium plurifarium *	Y14158	92	*Alphaproteobacteria *
R	ET-39	*Mesorhizobium loti *	HQ424911	88	*Mesorhizobium plurifarium *	Y14158	87	*Alphaproteobacteria *
S	ET-40	*Cupriavidus* sp.	AB266608	99	*Cupriavidus oxalaticus *	AF155567	98	*Betaproteobacteria *
S	ET-41	Unc. *Burkholderiaceae *	JF681924	95	*Thiobacter subterraneus *	AB180657	79	*Betaproteobacteria *
S	ET-42	Unc. *Bacteroidetes *	FM877553	87	*Ohtaekwangia koreensis *	GU117702	85	*Sphingobacteria *
S	ET-43	Unc. *Bacteroidetes *	FM877553	99	*Ohtaekwangia kribbensis *	GU117703	92	*Sphingobacteria *

Frac.: Fraction analysed; Unc.: Uncultured.
